# Mechanical and Microstructural Properties of Thermally Sprayed Metallic Materials in Compression Tests over a Vast Range of Strain Rates

**DOI:** 10.3390/ma16247566

**Published:** 2023-12-08

**Authors:** Artur Wypych, Tomasz Jankowiak, Wojciech Sumelka

**Affiliations:** 1 Institute of Materials Engineering, Poznan University of Technology, Jana Pawla II 24, 60-965 Poznan, Poland; artur.wypych@put.poznan.pl; 2 Institute of Structural Analysis, Poznan University of Technology, Piotrowo 5, 60-965 Poznan, Poland; wojciech.sumelka@put.poznan.pl

**Keywords:** thermal spraying of coatings, coating porosity, coating hardness, static strength of coatings, dynamic strength of coatings

## Abstract

This paper presents the mechanical behavior of thermally sprayed coatings produced using an arc wire coating material. The produced coatings were cut and subjected to strength resistance tests in static and in dynamic loading. The compressive behavior for the strain rates between 0.001 1/s and 2612 1/s was examined. The strain rate sensitivity of the material was recognized in the material during dynamic loading using the SHPB technique. Microstructural observations were made, and properties such as changes in porosity and the microhardness of the coatings tested were examined. A significant reduction in coating porosity was demonstrated after static loading (90%) and dynamic loading (86%). The result of porosity reduction is the strengthening of the coatings through an increase in the microhardness of these coatings after loading in the static test (160 HV 0.3/8) and the dynamic test (278 HV 0.3/8). As a result of the tests, the coatings retain their cohesion and remain consistent. At the same time, they can absorb a significant amount of mechanical energy due to plastic deformation and porosity reduction. The presented results concern a completely new coating material created from a core wire.

## 1. Introduction

Thermal spray coatings offer a wide range of advantages that can significantly benefit various industries. The motivation for this work comes from the fact that the use of thermally sprayed metallic materials in the industry may improve wear resistance, corrosion protection [1], dimensional restoration, or friction properties [2,3,4,5,6,7]. The versatility and applicability of thermal spray processes make them a valuable technology for improving performance and reducing costs in manufacturing equipment, processes, and the products produced [8]. The characteristics of materials, especially their mechanical attributes, are typically intricately linked to their microstructure. s. The interplay between microstructure and material properties constitutes a central focus within the field of materials science. This principle undeniably extends to thermally sprayed coatings as well [9]. Based on a literature analysis, the authors concluded that there are no studies at both static and dynamic properties of this type materials. Considering the coatings in question are used to protect elements loaded in this way, it was decided to carry out this type of research. Li and Ohmori (2002) investigated only quasi-static properties of coatings like, for example, the Young’s modulus and its connection to porosity [9]. McCoy and others (2020) analyzed the dynamic compressive properties of thermally sprayed materials only for high pressures [10]. They linked them also with the change in the microstructure of the coatings [10]. However, there are no scientific studies on the static and dynamic properties of coatings, which may be subject to various exceptional loads during operation. It has also not been investigated how the microstructure of coatings, including their porosity and hardness, changes under the influence of these loads. The methods of testing of typical metallic materials are known both in static and in dynamic - aspect [11,12,13,14,15].

This article analyzes the mechanical and internal structural properties of metallic coatings. These coatings are produced and applied by thermal (arc) spraying using coating materials in the form of wires. The discussed coatings can be used for additional bulletproof protection applied to existing structures while maintaining a relatively low weight. They can also be used in the energy or mining industry to cover parts of mineral processing machines.

Arc wire spraying is one option where the material of the wire is the primary source of the coating formed. In this technique, heat generation is achieved using an electric arc, employing two wires through which electric current flows. Herein, as these wires are brought closer together, the electric current forms a short circuit between them, resulting in a temperature of approximately 4000 °C. This elevated temperature causes the wire tips to melt. Once molten, either compressed air or an inert gas breaks down and accelerates the molten metal toward the surface [3,7]. The genetic algorithm optimization method was utilized by Swain and others 2022 [16] to achieve the ideal parameter configuration for the plasma spray coating procedure. From an application point of view, an important question arises: what are the mechanical and microstructural properties of such materials—especially over a vast range of strain rates?

To answer the above fundamental question, a comprehensive experimental study was elaborated—both from a microstructural and mechanical point of view. During the tests, it was determined how the properties of the material’s microstructure, such as porosity and hardness, change under the influence of static and dynamic loads. On the other hand, in the mechanical tests, strain–stress curves were analyzed. Samples in the form of cylinders with a diameter of 6 mm and a height of 3 mm were used. They were prepared and made in the Welding Laboratory of Poznan University of Technology, using a station to support thermal spraying processes. They were equipped with an AWS 400 Flame Spray Technologies spray device, a Kuka KR 16 manipulator, and a Nederman filter and ventilation system. During specimen preparation, the energy of the spray jet is regulated by changing the values of parameters such as the current (A), arc voltage (V), and pressure of the transporting gas (bar). A coating material as a core wire was applied. The wire characterizes the 1.6 mm diameter and a chemical composition contains the Fe–82, Cr–9, C–4, Si–2, O–3 in % weight. Materials of this type make it possible to produce coatings resistant to impact loads, as well as in the form of abrasion, and are characterized by a microhardness of 260 HV 0.3/8. The coatings were made on the substrate, from which they were cut off in the next treatment, and the material in the form of only the coating, i.e., without the steel substrate, was tested—hence, truly only the properties of the coating were tested. The mechanical properties of the thermally sprayed metallic materials are not yet recognized in the literature, especially in compression (both static and dynamic). It was vital that the samples were cut using a metallographic cutting machine with an adjustable feed speed and permanent liquid cooling. This eliminated uncontrolled stresses in the coating resulting from excessive pressure at too high a cutting speed and microstructural transformations leading to changes in the mechanical properties of coating due to heating during cutting.

Two series of experiments were prepared. The first used a hydraulic machine (static) and the second used a split Hokinson pressure bar (dynamic) [11,12,14,15,17]. The internal structure before and after the test was analyzed.

## 2. Experimental Set-Up and Test Description—Static and Dynamic Tests

### 2.1. General Remarks

Thermally sprayed parameters determine the energy of the spray jet. In the spraying process, the coating material is heated with arc heat at a temperature of about 6000 °C. As a result of the wires melting, droplets are formed, which are immediately removed from the melting zone by a stream of compressed gas. After exiting the nozzle of the spray gun, a spray jet is formed. The energy of this flux determines the micro-structure of the coating formed from the coating materials melted in the arc. The basic characteristics of coatings, such as porosity, are a function of the kinetic and thermal energy of particles formed in the spray jet. As a result of the collision of these particles with the ground, they are deformed, adapting to the shape of the ground on which they fall. With the increase in the kinetic energy of the spray jet, the contact surface of the particles with the substrate is larger, and the effect of matching individual particles to the substrate is better [16]. As a result of the set process parameters, it is possible to produce coatings with the expected porosity. In the experiment, the basic process parameters were used in the form of a current of 139 A, voltage was the 18.8 V, and working gas pressure was the 3.8 bar. The values of these parameters allowed us to obtain the initial porosity of the test coatings of 5.1%.

Porosity measurements were carried out using the optical method, using graphic software (Motic Image Plus v2.0, Xiamen, China), where photos with a resolution of 4.6 Mpix (1800 × 2700 pixels) taken on a Mira 3 (Tescan, Brno, Czech Republic) electron microscope were analyzed.

**Figure 1:**
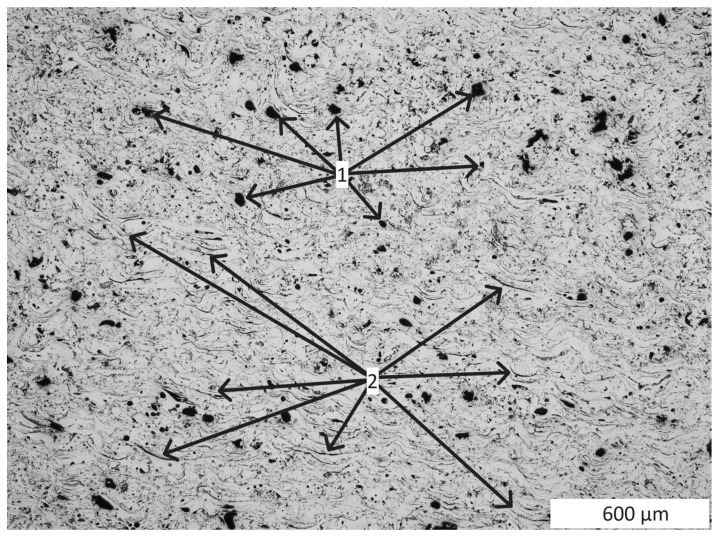
View of the microstructure of the cross-section of the prepared test coating. 1—porous, 2—oxide bands.

**Figure 2:**
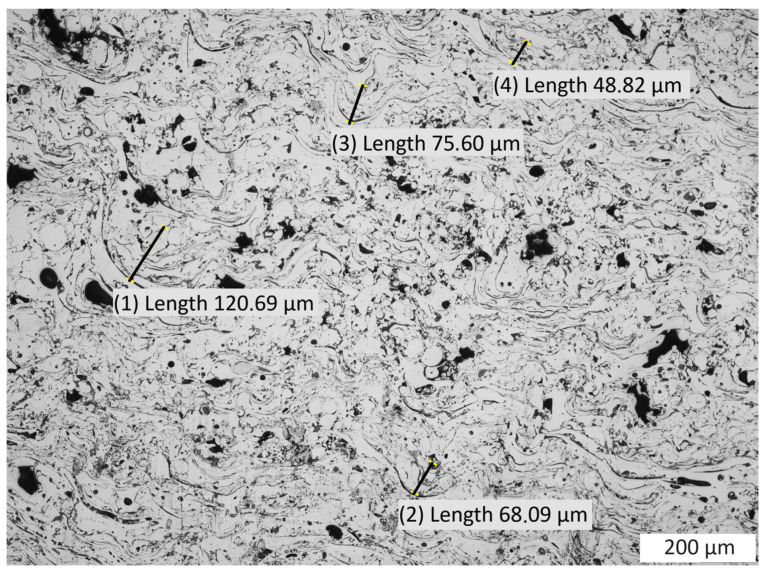
View of the coating microstructure with deformations of particles as a result of depositing on the substrate; the dimensions of deformation are from 48.82 µm to 120.69 µm.

The microstructure of the created test coating consists of porosities (1 in Figure 1) present in the amount of 5.1% and oxide bands shown on the coatings cross-sections (2 in Figure 1). The presence of these oxide bands is conducive to maintaining increased cohesion of the coatings due to their sliding function—they facilitate dislocation and reduce internal stresses in the coating. Due to the varied size of the particles of the coating material in the spray jet, their kinetic energy and the degree of deformation of these large particles are also differentiated. At the moment of impact with the ground, the size of these deformations ranges from almost 49 µm to over 120 µm (Figure 2) in the test coating prepared for the experiment. Sample preparation was carried out in accordance with the requirements of the ASTM E1920-97 standard [18].

Specimens in the form of cylinders with a diameter of 6 mm and a height of 3 mm were prepared for the tests. The ASTM standard was used in the testing procedure, taking into account the need to test very small samples with a thickness of 3 mm [19]. There is currently no existing ASTM standard for dynamic compression testing of metals for strain rates about 1000 1/s [20]. Samples were produced using a template where cylindrical elements of the coating material were obtained after spraying. In the following procedure, the cylindrical samples were planed with flat surfaces to ensure mutual perpendicularity of the cylinder basics planes to the axis of symmetry of the cylinders (see Table 1). The preparation of the samples ensured the elimination of the orthogonal components of the loading force and the shear effect during the tests.

The microstructure of the samples, containing both porosity and oxide bands and strongly deformed particles of the coating material, enabled the test samples to be formed into cylinders. Both, static and dynamic loads, cause significant microstructural deformations of coatings. The deformability of the coating is variable over time. In the first phase of loading, the porosity is reduced and dispersed, and dislocations in the coating volume are facilitated by the presence of oxide bands. After reducing the porosity, the coating properties are similar to solid material through strain hardening. Hence, in the first phase, the deformation is relatively large in relation to the load compared to the amount of deformation in the later stage of the test when loading the sample in a single test cycle.

**Figure 3:**
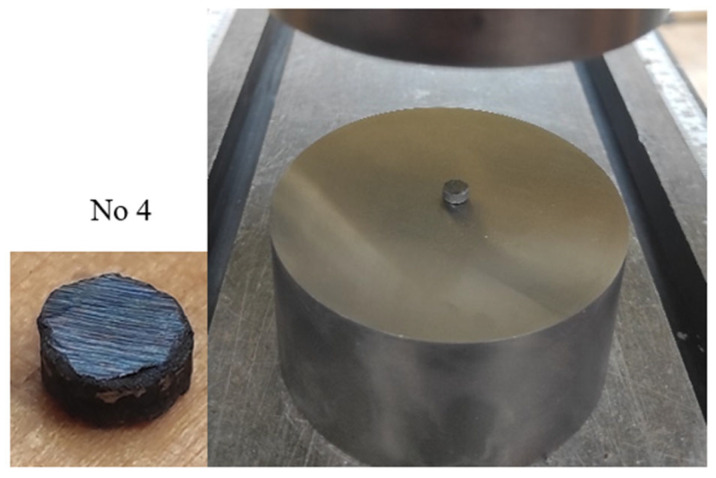
Geometry of a conical specimen with the view on the hydraulic machine platen (specimen No. 4).

Finally, the thermally sprayed metallic material is tested under static and dynamic compression. Two different experimental set-ups are used for both cases. The static tests are performed using a hydraulic machine (INSTRON 8505) with displacement control. The strain rate in these tests is around 0.001 1/s. As mentioned, cylindrical specimens with similar dimensions were used in all tests (diameter 6 mm and height 3 mm) (see Table 1). Before and after the tests, the microstructure of the specimens was analyzed. Before each test, the specimen geometry was measured, and the size was captured (in the static case, see Table 1). Additionally, specimen No. 4, located in the testing machine (platen) before the test, along with a close-up showing its exact shape and any irregularities, is presented in Figure 3. In the table and subsequent Equations (1)–(3), the heights of the samples are marked as $H_{s}$ and their diameters as $D_{s}$. During the test, the specimens are compressed kinematically by the pressure plates. The bottom plate is fixed, and the velocity of the top is 0.003 mm/s. It gives a strain rate equal to 0.001 1/s. The nominal strain $\varepsilon_{nom}$ and stress $\sigma_{nom}$ were calculated in each test based on the shortening of the specimen ∆H and compressive force P, using the following equation, Equation (1).

(1) $$\varepsilon_{nom}=\frac{∆H}{H_{s}}\;\mathrm{and}\;\sigma_{nom}=\frac{P}{\pi {\left(D_{s}/2\right)}^{2}}$$

The positive value for both quantities (∆H and P) in compression is assumed. Next, the true strain $\varepsilon_{true}$ and Cauchy stress $\sigma_{cauchy}$ were calculated using the following formula, Equation (2).

(2) $$\varepsilon_{true}=-\ln\left(1-\varepsilon_{nom}\right)\;and\;\sigma_{cauchy}=\sigma_{nom}\left(1-\varepsilon_{nom}\right)\;$$

These strain and stress measures were further used to calibrate the parameters of the constitutive model for the analyzed material.

**Figure 4:**
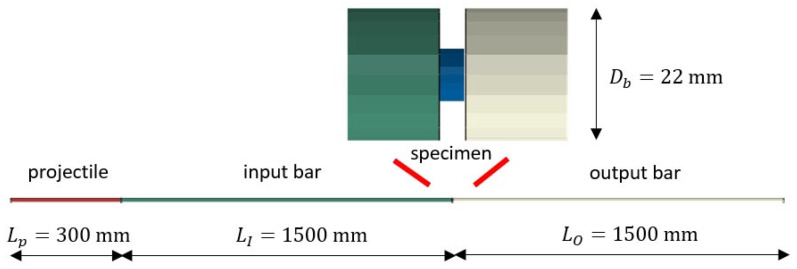
SHPB device used in dynamic compression.

The dynamic tests were performed using an SHPB device, as shown in Figure 4. The experimental set-up consists of a projectile and input and output bars between which a specimen of the material to be tested is placed [11,12,13,14,15].

**Figure 5:**
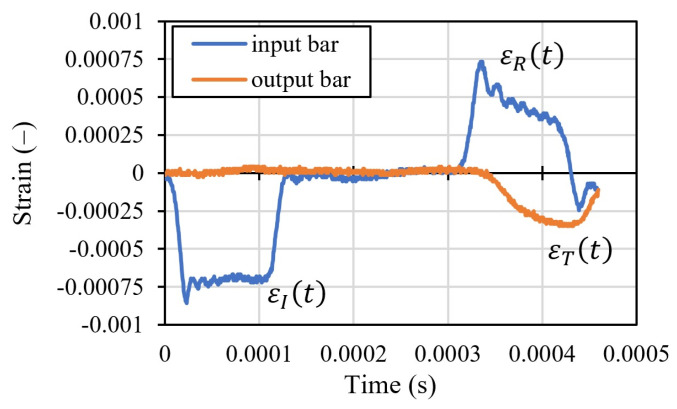
Typical curves recorded in input and output bar for initial velocity of the projectile equal to 7.32 m/s.

During the test, a projectile is accelerated by the gas gun for a given pressure. It then impacts the input bar with the velocity $V_{0}$. It induces a compressive incident wave of intensity ($\varepsilon_{I}\left(t\right)$ and $\sigma_{I}\left(t\right)$) in it. The wavelength ($t_{I}$) depends on the length of the projectile $L_{p}$ [12]. The wave propagates along the bar and reaches the specimen by compressing it. The wave then passes into the output bar as a compressive transmitted wave ($\varepsilon_{T}\left(t\right)$ and $\sigma_{T}\left(t\right)$) and, due to mechanical impedance, reflects and propagates in the return direction as a tensile reflected wave ($\varepsilon_{R}\left(t\right)$ and $\sigma_{R}\left(t\right)$). According to the dynamic equilibrium of the system, the three waves, (Figure 5), are in the relation $\varepsilon_{I}\left(t\right)=\varepsilon_{T}\left(t\right)-\varepsilon_{R}\left(t\right)$. The following equations are used to calculate the stress σ, strain rate $\dot{\varepsilon }$, and strain ε of the specimen during each test, Equations (3)–(6).

(3) $$\sigma \left(t\right)=E_{b}{\left(\frac{D_{b}}{D_{s}}\right)}^{2}\left|\varepsilon_{T}\left(t\right)\right|$$

(4) $$\dot{\varepsilon }\left(t\right)=\frac{2C_{0}}{L_{0}}\left|\varepsilon_{R}\left(t\right)\right|$$

(5) $$\varepsilon \left(t\right)=\frac{2C_{0}}{L_{0}}x\int_{0}^{t}\left|\varepsilon_{R}\left(x\right)\right|dx$$

Finally, after calculating the mean strain rate ${\dot{\varepsilon }}_{mean}$, the thermo-visco-plastic behavior of the material $\sigma \left({\bar{\varepsilon }}_{p},\;{\dot{\bar{\varepsilon }}}_{p},\;T\right)$ can be determined [12,13,21]. This is the strain hardening and strain rate hardening function. The arguments of that function are the equivalent plastic strain ${\bar{\varepsilon }}_{p}$ and strain rate ${\dot{\bar{\varepsilon }}}_{p}$ variables. The current experiments were performed at room temperature, 20 °C. The strain rate depends on the projectile’s initial velocity, $V_{0}$. In the current tests, the strain rate was between 1500 1/s and 8000 1/s. The typical waves recorded directly in the currently analyzed tests, for a projectile velocity of 7.32 1/s, are presented in Figure 5. The incident $\varepsilon_{I}$, reflected $\varepsilon_{R}$, and transmitted $\varepsilon_{T}$ waves are visible. The curves present the strain intensities recorded during a test in the middle of the bars using full bridge gauges.

**Figure 6:**
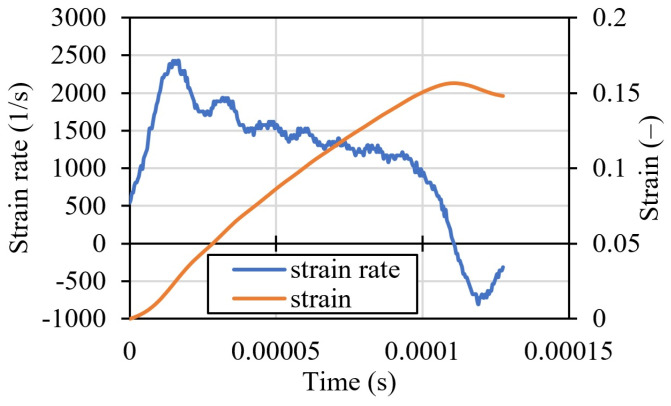
Strain rate and strain in the specimen for an initial velocity of the projectile equal to 7.32 m/s.

Using Equations (4) and (5), the plot in Figure 6 was created. The analysis of the waves was conducted in SciLab v6.1.0 software using our own code. The strain rate in the specimen for a projectile initial velocity of 7.32 m/s varies in time during the compression test. The average strain rate ${\dot{\varepsilon }}_{mean}$ for this test was ~1500 1/s. The final deformation of the specimen calculated from the waves was ~0.15 (nominal strain). The unloading of the specimen is also observed in Figure 6.

### 2.2. Static Tests

**Figure 7:**
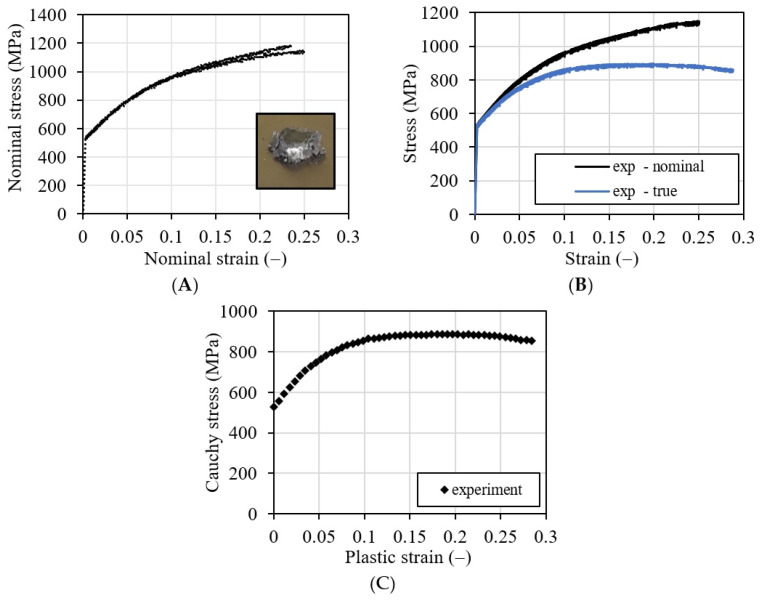
Results of the static experimental tests (strain rate 0.001 1/s); (**A**) nominal stress–strain curve, (**B**) nominal versus true curve, (**C**) strain hardening function.

The compressive tests [12] were performed for all specimens (four specimens). After each test, the curve force-displacement (P−∆H) was analyzed. First of all, the nominal strain and nominal stress were calculated using Equation (1) (see Figure 7A). Next, the true stress–strain curve was presented together with the nominal one in Figure 7B. The last stage of the identification was to define the strain hardening in the quasi-static case for a strain rate of 0.001 1/s. At the end, the small elastic strain is subtracted from the total strain, and finally, the plastic strain was calculated. The final plot is presented in Figure 7C.

**Figure 8:**
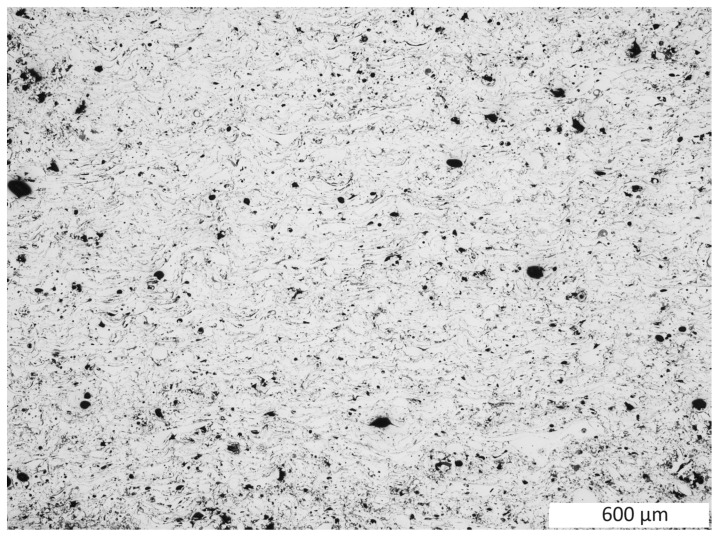
View of the microstructure of the cross-section of the static compression test coating.

**Figure 9:**
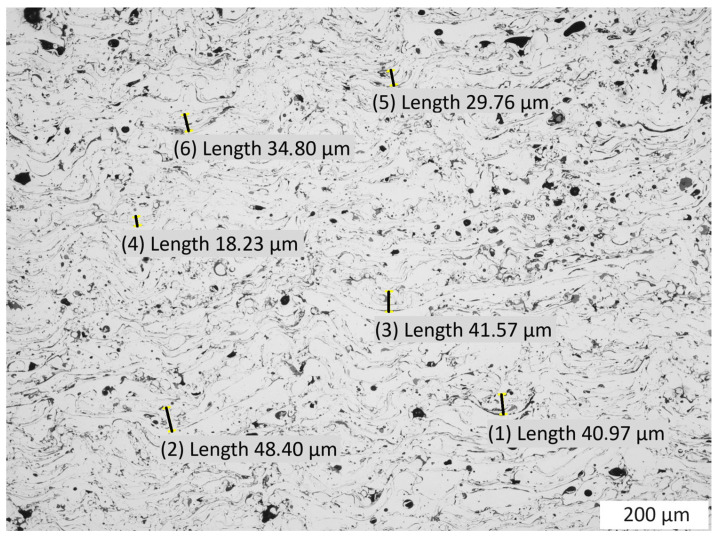
View of the coating microstructure with deformations of particles as a result of the static compression load of the specimen; the dimensions of deformation are from 18.23 µm to 48.40 µm.

As a result of the static loading and deformation in the test samples, a significant reduction in porosity was observed, which in this case is 0.5%. The oxide bands were significantly deformed, facilitating dislocations in the coating. The magnitude of these deformations is visible through the reduction in curvatures created during the production of the test sample (Figure 1, Figure 8 and Figure 9). As a result of the deformation of the coating material particles in the coating due to the static load, these deformations range from 18.23 µm to 48.40 µm. Due to the slipping mechanism using oxide bands, the sample retained cohesion throughout the test and after completion. As a result of the static load, the coating was strain strengthened, leading to an increase in the average hardness; the value after the static load test is 420 HV 0.3/8.

### 2.3. Dynamic Tests

**Figure 10:**
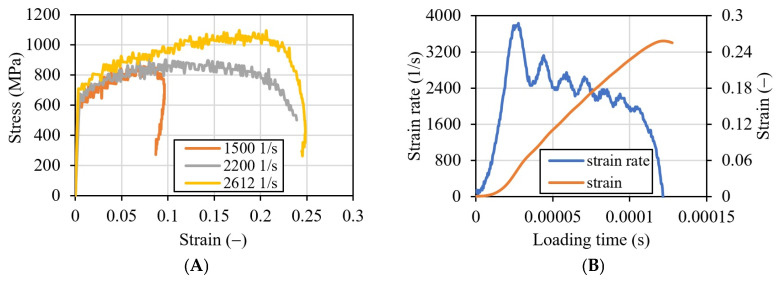
Results of the dynamic compression experimental tests using SHPB; (**A**) true stress–strain curves for three strain rates; (**B**) strain rate and strain curves versus loading time for impact velocity 11.052 m/s (average strain rate during the impact: 2612 1/s).

Dynamic compressive tests for different impact velocities were performed (nine specimens—three for each velocity). All the specimens were tested and, finally, the average values from the curves were calculated for each strain rate. Final true stress–strain curves are plotted for each average strain rate in Figure 10A. The average strain rates were calculated taking into account that these values vary during dynamic compression (Figure 10B). Here, the strain rate was calculated as 2612 1/s. In Figure 10B, strain history in the specimen during dynamic compression is also calculated (Equation (5)). The curve for the strain rate for an impact velocity of 7.32 m/s is presented in Figure 6. The average strain rate for these tests is 1500 1/s. In Figure 10B, the same curve is presented for a velocity of 11.052 m/s. The true stress–strain curve after the test was recalculated for the strain hardening function $\sigma \left({\bar{\varepsilon }}_{p},\;{\dot{\bar{\varepsilon }}}_{p},\;T\right)$.

The positive strain rate sensitivity is visible during the impact tests (using SHPB), especially if the results are compared with the quasi-static ones (c.f. Section 2.2).

**Figure 11:**
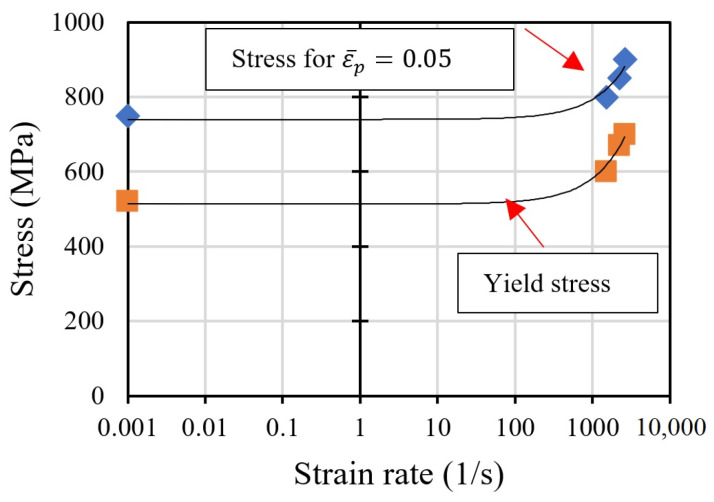
Strain rate sensitivity calculated in static and dynamic tests.

As a result of the presented experimental studies, the strain rate sensitivity of the material was observed (Figure 11). The nonlinear trend in the logarithmic scale of the strain rate is shown.

**Figure 12:**
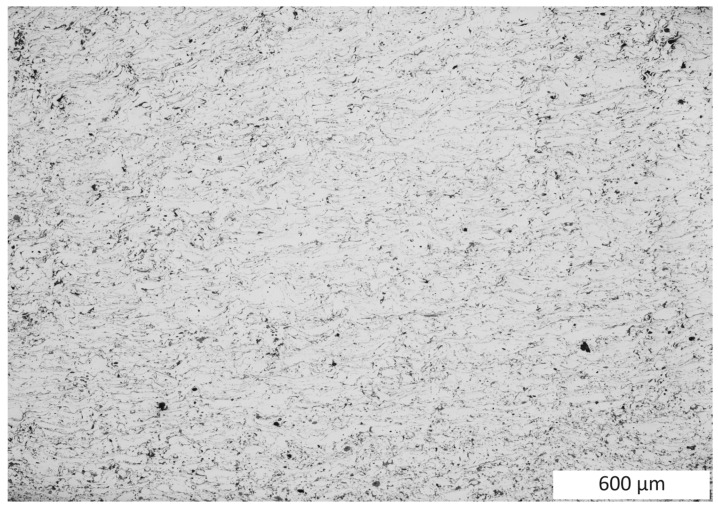
View of the microstructure of a cross-section of the dynamic compression test coating.

**Figure 13:**
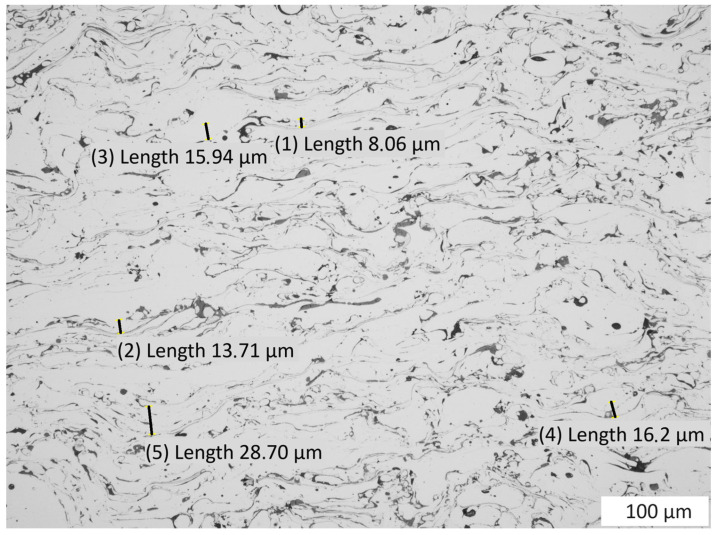
View of the coating microstructure with deformations of particles as a result of the dynamic compression load of the specimen; the dimensions of deformation are from 8.06 µm to 28.70 µm.

Changes in the material structure were observed. As a result of the dynamic load, the porosity in the coating was reduced by 0.7% after the test. The load dynamics led to a significant dispersion of very small porosities and fragmentation of their oxide bands due to the strong deformation of the coating material particles in the coating, the size of which ranged from 8.06 to 28.70 (Figure 12 and Figure 13). As a result of these strong deformations resulting from the sudden load, the test material was strengthened, where the hardness increased to an average value of 538 HV 0.3/8.

### 2.4. Evolution of the Microstructure

**Figure 14:**
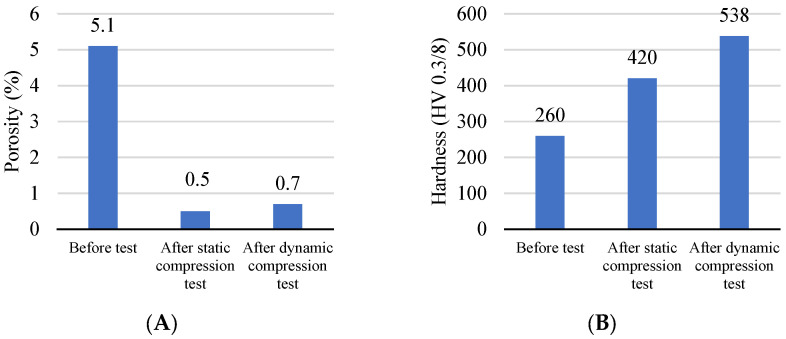
Change in: (**A**) porosity and (**B**) hardness of coating as a result of deformation.

During static and dynamic compression, coating materials undergo distinct changes in their microstructure, influencing both porosity and hardness. The following trends are observed:

- A decrease in porosity was observed due to both static and dynamic interactions (Figure 14A). The initial porosity of 5.1% decreased to 0.5% (by 90%) in the case of static compression and to 0.7% (by 86%) in the dynamic case.
- An increase in hardness was observed as a result of both static and dynamic interactions (Figure 14B). The initial hardness of 260 HV 0.3/8 increased to 420 HV 0.3/8 (by 62%) in the case of static compression and to 538 HV 0.3/8 (by 107%) in dynamic compression.

## 3. Conclusions

Thermal arc spraying of coatings can be used to produce coatings with a high ability to absorb static and dynamic energy. Energy absorption is achieved by reducing the porosity and plastic deformation of a coating during loading. At the same time, this coating is strengthened by significantly hindering dislocations in the coating due to plastic deformation and filling of the pores with the coating material.

This work examined how the material properties, including porosity and hardness, change during static and dynamic compression. Unique qualitative and quantitative results were obtained in this area. A key result of the research is also obtaining information on the mechanical behavior of the material. A stress–strain curve was determined for quasi-static deformation rates. It also turned out that the coating material is sensitive to deformation rates. The stress level in the case of dynamic loads is higher than that in the case of quasi-static loads. Therefore, the material is highly sensitive to deformation speed.

In view of further plans for coatings, research will be carried out on changes in the properties of coatings under load as a function of the change in the energy of the spray stream and the kinetic energy of particles in this. To control the kinetic energy of particles in the spray stream, high-speed cameras will be used with recording speeds up to 1 × 10^6^ frames per second. In this way, the size and speed of particles and, indirectly, the porosity and oxidation degree of the coatings will be controlled. The produced test coatings will be checked again under different loading conditions, where the measurement will provide information on the change in strength properties as a function of the morphology of the arc thermally sprayed coatings.

## Figures and Tables

**Table 1:** The dimensions and geometry of the conical specimens in static cases.

	**No. 1**	**No. 2**	**No. 3**	**No. 4**	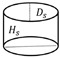
$D_{s}$ [mm]	6.06	5.97	6.00	6.08	
$H_{s}$ [mm]	2.85	2.93	2.99	3.07	

## Data Availability

Data are contained within the article.
